# Whole genome sequencing in paediatric channelopathy and cardiomyopathy

**DOI:** 10.3389/fcvm.2024.1335527

**Published:** 2024-03-20

**Authors:** Sit Yee Kwok, Anna Ka Yee Kwong, Julia Zhuo Shi, Connie Fong Ying Shih, Mianne Lee, Christopher C. Y. Mak, Martin Chui, Sabrina Tsao, Brian Hon Yin Chung

**Affiliations:** ^1^ Department of Paediatrics and Adolescent Medicine, Hong Kong Children’s Hospital, Hong Kong, Hong Kong SAR, China; ^2^ Department of Paediatrics and Adolescent Medicine, School of Clinical Medicine, The University of Hong Kong, Hong Kong, Hong Kong SAR, China; ^3^ Clinical Genetics Service Unit, Hong Kong Children’s Hospital, Hong Kong, Hong Kong SAR, China

**Keywords:** channelopathy, cardiomyopathy, paediatrics, genome sequencing, intronic variants, MYBPC3, Barth syndrome, RASopathy

## Abstract

**Background:**

Precision medicine in paediatric cardiac channelopathy and cardiomyopathy has a rapid advancement over the past years. Compared to conventional gene panel and exome-based testing, whole genome sequencing (WGS) offers additional coverage at the promoter, intronic regions and the mitochondrial genome. However, the data on use of WGS to evaluate the genetic cause of these cardiovascular conditions in children and adolescents are limited.

**Methods:**

In a tertiary paediatric cardiology center, we recruited all patients diagnosed with cardiac channelopathy and cardiomyopathy between the ages of 0 and 18 years old, who had negative genetic findings with prior gene panel or exome-based testing. After genetic counselling, blood samples were collected from the subjects and both their parents for WGS analysis.

**Results:**

A total of 31 patients (11 cardiac channelopathy and 20 cardiomyopathy) were recruited. Four intronic splice-site variants were identified in three cardiomyopathy patients, which were not identified in previous whole exome sequencing. These included a pathogenic variant in *TAFAZZIN:c.284+5G>A* (Barth syndrome), a variant of unknown significance (VUS) in *MYBPC3:c.1224-80G>A* and 2 compound heterozygous LP variants in *LZTR1* (*LZTR1:c.1943-256C>T* and *LZTR1:c1261-3C>G*) in a patient with clinical features of RASopathy. There was an additional diagnostic yield of 1.94% using WGS for identification of intronic variants, on top of conventional gene testing.

**Conclusion:**

WGS plays a role in identifying additional intronic splice-site variants in paediatric patients with isolated cardiomyopathy. With the demonstrated low extra yield of WGS albeit its ability to provide potential clinically important information, WGS should be considered in selected paediatric cases of cardiac channelopathy and cardiomyopathy in a cost-effective manner.

## 1 Introduction

Precision medicine has revolutionized the field of cardiology, leading to significant changes in practice. It plays a pivotal role in guiding prognostic and therapeutic decision-making, as well as in preventing sudden cardiac death (SCD) in asymptomatic family members. These conditions encompress cardiomyopathies and cardiac channelopathies. Panel sequencing and exome-based genetic testing have been widely utilized to identify the molecular aetiology of these cardiovascular conditions in recent years. However, these approaches have limitations in their coverage, particularly in non-coding regions, deep intronic regions and the mitochondrial genome. Whole genome sequencing (WGS), on the other hand, offers a more comprehensive coverage of these regions. The use of WGS to evaluate the genetic cause of cardiovascular conditions in children and adolescents is limited. Bagnall et al. conducted WGS on 58 unrelated patients with hypertrophic cardiomyopathy (HCM) and identified five patients with pathogenic variants in the non-coding genome, which were missed by gene panel or whole exome sequencing (WES) (1). This underscores the significant potential of WGS in identifying the underlying genetic causes in these diseases.

As the sole tertiary paediatric cardiology center in Hong Kong, all cases of advanced inherited cardiovascular conditions are referred to and managed in our center. Our experience in the management using precision medicine has been reported, including long QT syndrome (LQTS), catecholaminergic polymorphic ventricular tachycardia (CPVT), *SCN5A* mutation and cardiomyopathy (2–5). This study aims to share our initial experience in utilizing WGS to unravel the molecular etiology in a 15-year cohort of paediatric cardiomyopathy and cardiac channelopathy.

## 2 Materials and methods

### 2.1 Patient recruitment and clinical data collection

All patients with a diagnosis of cardiac channelopathy and primary cardiomyopathy over the past 15 years (January 2007–December 2021) have undergone either target gene panel testing using Sanger sequencing/multiplex-ligation dependent probe amplification (MLPA)/next generation sequencing or WES in Department of Paediatric Cardiology, Queen Mary Hospital, Hong Kong as previously reported (2, 3, 5). The service was subsequently relocated to Hong Kong Children's Hospital. A comprehensive database was maintained to track all patients who underwent genetic testing at our center. The following patients in the database were recruited:

1. Patients diagnosed with cardiac channelopathy and cardiomyopathy between the ages of 0 and 18 years old, meeting the diagnostic criteria of the two conditions (6–8),

AND

2. Patients who did not receive a positive genetic diagnosis from their prior genetic investigations relating to the phenotype. Patients with VUS in genes with no definite or moderate evidence were also included.

If the patients were found to have variants of uncertain significance (VUS), a thorough review of the gene information was conducted. The gene-disease validity was assessed based on expert recommendation by ClinGen (https://www.clinicalgenome.org/). If the VUS genes were found to have a definitive or moderate evidence as genotype explanation for the concerned phenotype (i.e., VUS with clinical relevance), those patients were excluded from recruitment for WGS. Individuals with cardiomyopathy secondary to chemotherapy, myocarditis, or environmental toxin exposure or nutrient deficiency were excluded.

Eligible patients meeting the recruitment criteria were contacted and invited to participate in the study. Interviews were conducted with the patients and/or their parents, and pre-test genetic counselling was provided. Upon obtaining informed consent, blood samples were collected from the subjects and their parents. The following data were collected: demographic information, diagnosis, clinical history, pedigree and initial genotype information.

#### 2.1.1 Whole-genome sequencing

Genomic DNA was extracted from peripheral blood using Flexigene DNA Kit (Qiagen GmbH, Germany) and 550 ng of extracted DNA was fragmented to a peak size of around 300 bp for library preparation using KAPA Hyper Prep Kit (Roche, Switzerland). The fragmented DNAs underwent end-repair, A-tailing at the 3'end, adaptors ligation at the terminal ends with xGen® Dual Index UMI Adapters system (Integrated DNA Technologies, US). The adapter ligated library with size range 300–750 bp were selected by dual SPRI methods. The PCR-free libraries were denatured and diluted to optimal concentration for Pair-End 151 bp sequencing in Illumina NovaSeq 6,000 platform (Illumina, US).

#### 2.1.2 Quality control and data processing for whole-genome sequencing data

Using Illumina software bcl2fastq, sequencing reads were assigned into individual samples with sample. An average of 92%–93% of the bases achieved a quality score of Q30, indicating a base call accuracy of 99.9%. Fastq file were processed using DRAGEN Germline (v3.9.5) pipeline of BaseSpace (Illumina, USA) to map and align to hg19/GRCh37 human reference genome. The resulting BAM files were then input into the Illumina Dragen Joint Genotyping Pipeline to generate a joint call VCF for the detection of single nucleotide variant (SNV), insertion-deletion (indel), copy number variations (CNVs) and structural variations (SVs).

Variant call format (VCF) output was annotated and filtered using Geneyx Analysis platform (Geneyx Genomex Ltd., Israel, http://www.geneyx.com) according to the phenotypic association, allele frequencies in control population, deleteriousness by in silico prediction, segregation, and functional consequence. The main disease databases used include ClinVar, OMIM and publications, and the reference population databases were gnomAD, ESP, dbSNP and 1000G databases. The CNV and SV VCF outputs were annotated using AnnoSV1, incorporating the relevant Human Phenotype Ontology (HPO) for each case. The prioritization of candidate genes involved a gene panel specific to cardiomyopathy (*n* = 29) curated by the American College of Medical Genetics and Association of Molecular Pathology (ACMG/AMP) (9), as well as expanded cardiovascular disease gene panel (*n* = 217) adopted from the United Kingdom National Health Service (NHS), GeneDx, and Blueprint Genetics, which included primary cardiomyopathy and cardiac channelopathy genes (Supplementary Table S2). Open-genome examination was performed if no significant finding could be identified from the prioritized gene panels.

#### 2.1.3 Variant classification

Variants were scored according to the ACMG/AMP pathogenicity guidelines (10). The evidence of pathogenicity and benign impact was also influenced by the SVI General Recommendations for Using ACMG/AMP Criteria (https://clinicalgenome.org/working-groups/sequence-variant-interpretation/) as suggested by the ClinGen team (11–19). Additionally, ClinGen created a cardiomyopathy variant curation expert panel with a modified ACMG/AMP guideline framework for MYH7. If the mutation fell upon the MYH7 gene, the ClinGen curated variation classification was used (20). Similarly, a RASopathy variant curation expert panel with a modified framework for nine RASopathy genes was employed (21). As more variant expert panels had been released with modified ACMG/AMP guidelines for cardiomyopathy and cardiac channelopathy, the most updated version was used for analysis. In addition, Association for Clinical Genomic Science (ACGS) best practice guidelines was adopted for variant classification in some cases (22).

#### 2.1.4 Genetic test result validation and interpretation

SNVs and small indels were further validated using Sanger sequencing following standard protocol. For variants predicted to affect splicing, blood RNA sequencing was performed as previously described to provide functional evidence (23). The genetic test results were discussed by the multi-disciplinary team comprising cardiologists, clinical geneticists, genome analysts, and bioinformaticians. Post-test counselling was provided by geneticists or genetic counsellors. Variants that were not listed in GnomAD v.2.1.1 were referred to as “novel” throughout the manuscript.

### 2.2 Statistical analysis

Descriptive statistics were analyzed using SPSS and Excel. Continuous data were expressed as mean and standard deviation. A *p*-value of <0.05 was considered statistically significant.

## 3 Results

### 3.1 Results of recruitment

There was a total of 88 and 67 patients with diagnosis of channelopathy and cardiomyopathy respectively, who underwent genetic testing since 2007 in our cohort. Among them, 15 cardiac channelopathy and 36 cardiomyopathy patients underwent whole exome sequencing as their first genetic testing. Pathogenic or likely pathogenic variants were identified in 52 (59.1%) channelopathy patients and 32 (47.8%) cardiomyopathy patients. VUS in relevant/established disease genes were identified in 14 (15.9%) channelopathy patients and 9 (13.4%) cardiomyopathy patients.

**Figure 1:**
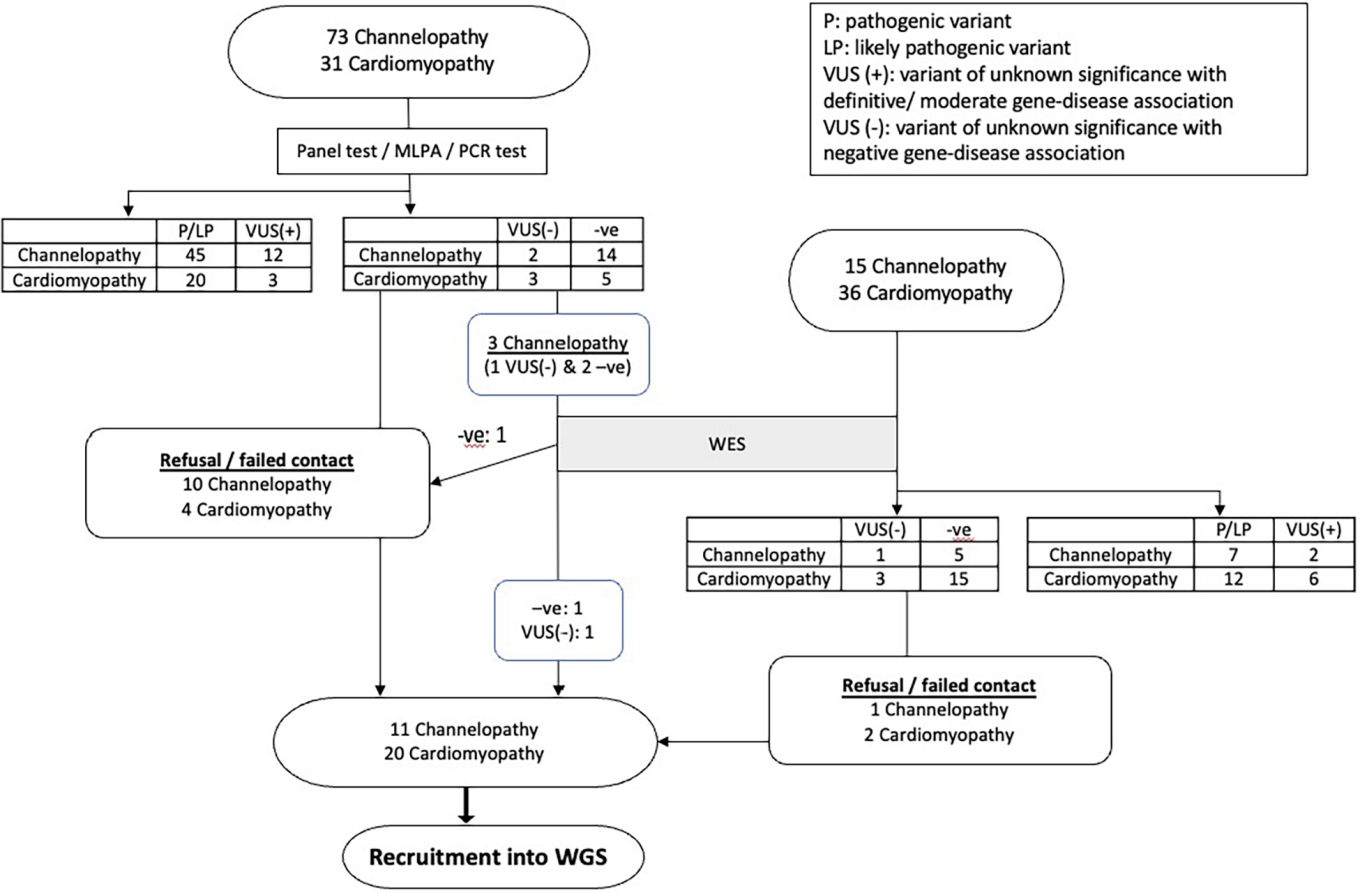
The flowchart summarizes the result of study recruitment of paediatric cardiac channelopathy and cardiomyopathy patients.

Among the remaining patients, 3 channelopathy and 6 cardiomyopathy patients had VUS with no gene-disease association, while 19 channelopathy patients and 20 cardiomyopathy patients had no variants identified in initial genetic testing. Figure 1 showed the detailed flow chart of our study recruitment.

A total of 48 patients (22 channelopathy and 26 cardiomyopathy) were eligible for recruitment into the study. However, 17 patients could not be recruited due to refusal or failure to be reached. Ultimately, 31 patients were recruited, and among them, 8 patients had VUS of low clinical relevance.

Among the 31 recruited subjects, 19 were male (61.3%). Their ages ranged from 9 months to 29 years old, with an average age of 14.36 ± 7.16 years. All patients were of Chinese ethnicity. Before enrolment, 22 patients had undergone WES testing. Table 1 presents the phenotype of the subjects and the genetic findings identified by WGS analysis. In total, 7 new clinical relevant variants could be identified in 6 patients. Four variants (in 3 patients) were intronic splice error variants, while 3 variants were identified in exome region. The use of WGS in our cohort resulted in an additional yield of 3.87% (1.94% for intronic variants) in the identification of clinically relevant genetic variants, in additional to conventional gene testing. The details of these 6 patients are described below:

**Table 1:** Phenotype of recruited subjects and results of whole genome sequencing.

Phenotype	Recruited subjects	Subjects with previous WES	Genes identified by prior testing, classified as VUS with limited gene-disease association)	Patient number (positive testing using WGS)	Variants identified by WGS	Pathogenicity	ACMG criteria used
DCM	8	8	*KCNH2, CBL, DSP*	*1*	*TAFAZZIN:c.284+5G>A*	P	PVS1, PP4_strong, PM2_supporting
			* *	*2*	*MYBPC3:c.1224-80G>A*	VUS	PVS1_moderate, PM2_supporting
HCM	6	5	* *	*3*	*LZTR1:c.1943-256C>T*	LP	PVS1, PM2_supporting, PM3
			* *	* *	*LZTR1:c1261-3C>G*	LP	PVS1, PM2_supporting, PM3
RCM	2	2	* *	* *	* *		
LVNC	3	1	*ACTN2, MT-TK*	*4*	*MYH7:c.1118C>A*	LP	PS2, PM2_supporting, PP3
ACM	1	0	*MYOM1/MYH11*	* *	* *		
LQTS	2	1	* *	* *	* *		
CPVT	2	0	* *	*5*	*RYR2:c.6679G>A*	LP	PM2_supporting, PP4, PM1_strong
			* *	*6*	*RYR2:c.1858T>G*	VUS	PM2_supporting, PP3, PP4
BrS	2	1	*AKAP9*	* *	* *		
SQTS	1	1	* *	* *	* *		
Idiopathic VT/VF	3	2	*VCL/KCNA5*	* *	* *		
SND	1	1	* *	* *	* *		
Total	31	22	* *	* *	* *		

DCM, dilated cardiomyopathy; HCM, hypertrophic cardiomyopathy; RCM, restrictive cardiomyopathy; LVNC, left ventricular non-compaction cardiomyopathy; ACM, arrhythmogenic cardiomyopathy; LQTS, long QT syndrome; CPVT, catecholaminergic polymorphic ventricular tachycardia; BrS, brugada syndrome; SQTS, short QT syndrome; VT/VF, ventricular tachycardia/ventricular fibrillation; SND, sinus node dysfunction; LP, likely pathogenic; P, pathogenic; VUS, variant of unknown significance.

#### 3.1.1 Patient 1—dilated cardiomyopathy (DCM) and Barth syndrome

**Figure 2:**
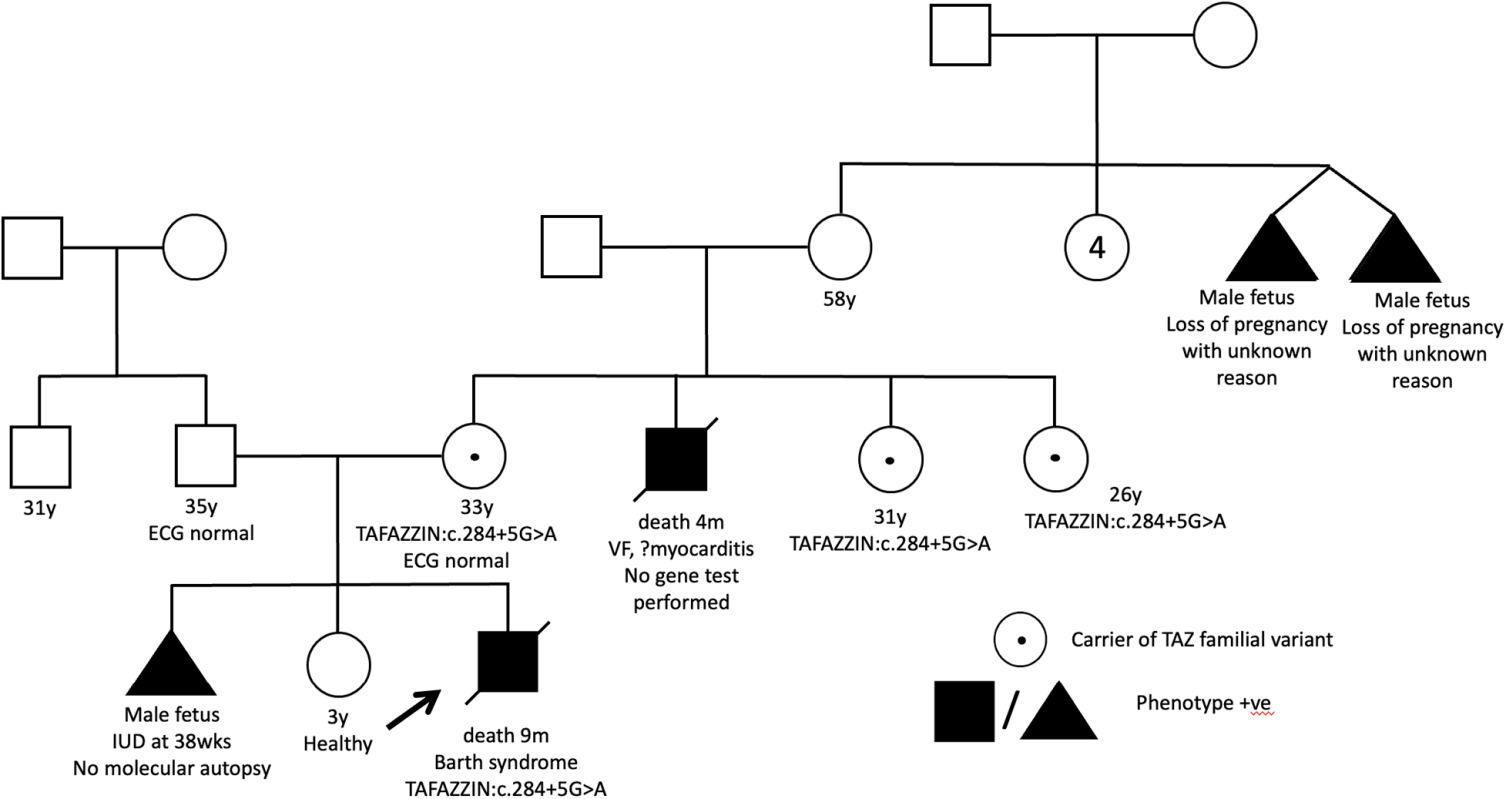
Pedigree of patient 1 showed multiple affected maternal male family members. His mother is a carrier of the familial variant.

A full-term male infant with a birth weight of 2.4 kg was born. The antenatal morphology scan revealed cardiomegaly and pericardial effusion. He had a strong family history of intrauterine and early infant death among male family members (Figure 2). At 2 weeks of age, he developed cardiogenic shock. Echocardiography confirmed the diagnosis of DCM with global left ventricular dysfunction. Additionally, he experienced neonatal ketotic hypoglycemia, evolving generalized hypotonia and gross motor delay. Initial metabolic investigations, including urine methylglutaric acid, were unremarkable. Genetic testing using WES only identified a variant in the *SCN5A* gene (*SCN5A: c.874G>A, p.Gly292Ser*) but the variant frequency in the East Asian population (8/19536, *f = *0.0004095, GnomAD) was too high to be considered significant for reporting. There were no other supporting in-silico or functional data to indicate its pathogenicity. No other variants of interest could be identified. Unfortunately, the patient succumbed to refractory heart failure at 9 months of age, and neutropenia was also observed.

**Figure 3:**
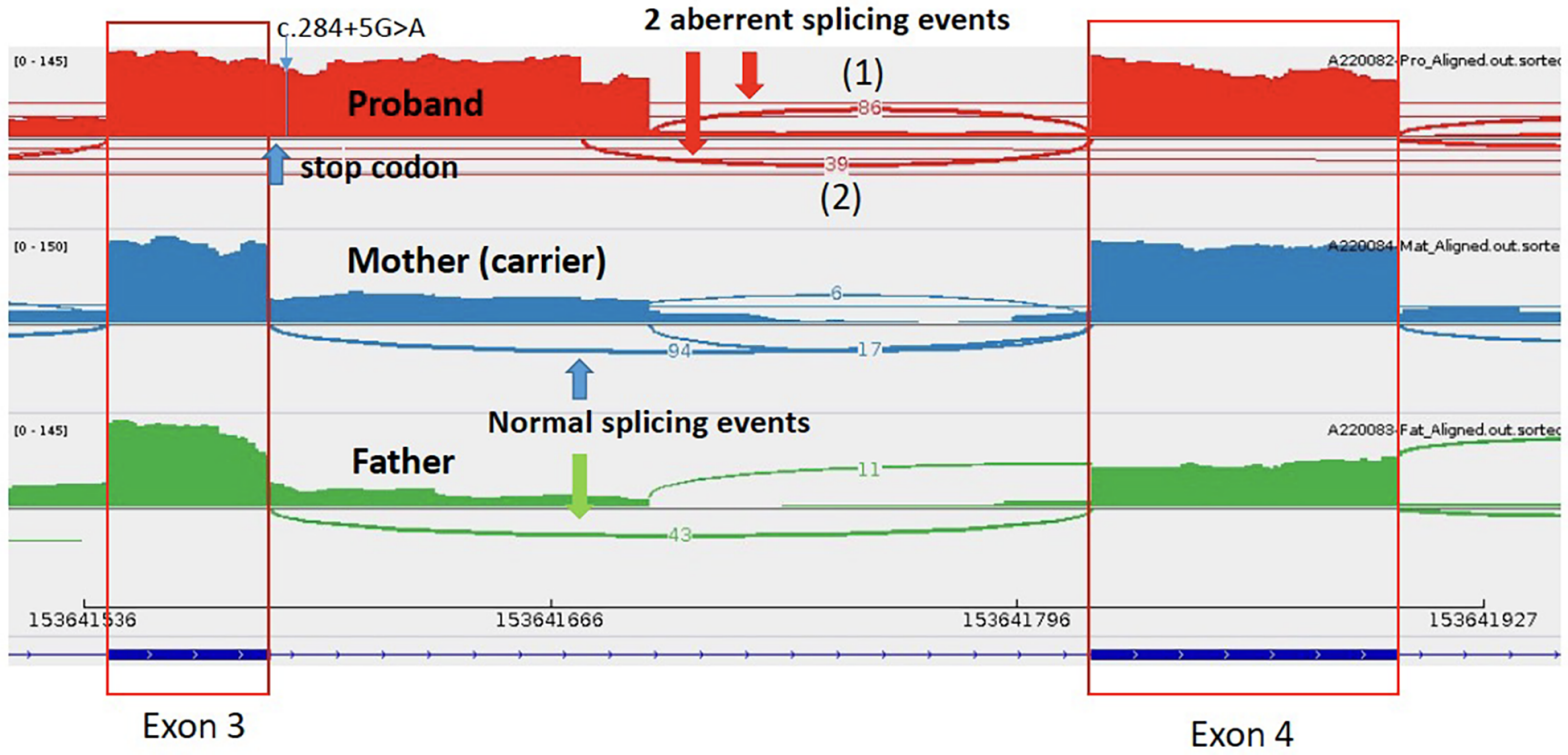
Mutation in the *TAFAZZIN* gene *(NM_000116.5):c.284+5G>A* in patient 1- two aberrant splicing events occur in proband, resulting in a 106 bp and another 87 bp intron retention, with an immature stop codon created at the start of intron 3.

Through WGS, a hemizygous +5 splice variant in the intronic region of chromosome X-153641594-G-A was identified in the *TAFAZZIN* gene *(NM_000116.5):c.284+5G>A*, inherited from the patient's mother. This variant was absent in the GnomAD normal population dataset. *In-silico* splice-site prediction tools predicts abnormal splicing (SpiceAI score was 0.81), suggestive of a cryptic splice variant (delta score ≥0.8). Using RNA-sequencing, the aberrant splicing event in the *TAFAZZIN* gene was detected as significant splicing outlier in the DROP pipeline, with a FDR = 0.016047. Integrated Genome View (IGV) displayed two aberrant splicing events occur in proband, resulting in a 106 bp and another 87 bp intron retention (Figure 3). An immature stop codon is created at the start of intron 3, predicted to form truncating and non-functional protein products. Repeated metabolic work-up including urine organic acid showed hyperexcretion of 3-methylglutaric acid and 3-methylglutaconic acid. Further cardiolipin analysis demonstrated elevated monolysocardiolipin/cardiolipin (MLCL/CL) ratio on dried bloodspot and lymphocytes which confirmed the diagnosis biochemically. According to ACMG and ACGS guidelines, the variant is classified as pathogenic (PVS1, PP4_strong and PM2_supporting).

#### 3.1.2 Patient 2—heart transplanted DCM and intronic MYBPC3 variant

The boy presented at his age of 12 years with exercise intolerance and echocardiography revealed DCM with severely depressed biventricular function. No secondary causes for his condition could be identified. Consequently, heart transplantation was deemed necessary for decompensated heart failure. The family history was unremarkable and previous genetic testing using WES yielded negative results.

Through WGS, an intronic variant in the *MYBPC3* gene (*NM_000256.3:c.1224-80G>A*) was identified. This specific intronic region was not adequately covered in good depth in previous WES analysis. The variant was inherited from his father and was rare in population (allele frequency = 0.0000637). Using SpliceAI, the variant received a high score, indicating its potential to cause aberrant splicing. Notably, this cryptic splice-altering variant in *MYBPC3* has also been observed in two HCM patients in which an exonization of the last 78 bp of intron 13 in the mutated transcript, was demonstrated by minigene splicing report assay. This exonization results in an insertion of 26 amino acids (24, 25). A case with the same variant was also reported in ClinVar, where it was classified as a likely pathogenic (LP) variant, albeit without accompanying phenotype information. *MYBPC3* is a gene with a definitive evidence of HCM but with a limited evidence of DCM association. Although the gene mutation has been reported to cause DCM (26), there is still a lack of robust evidence in this gene-disease association. Complexities exist in genotype-phenotype relationships, in relating MYBPC3 variant change in causing dilated cardiomyopathy. The patient might suffer from burnt-out HCM but it could not be proven. As biallelic variants in *MYBPC3* are associated with severe early onset disease (27), a second hit in this gene was reanalyzed again with no additional findings. The variant was classified as VUS based on the ACMG guidelines (PVS1_moderate, PM2_supporting). His father's cardiovascular investigation was normal with no other clinically affected paternal family members.

#### 3.1.3 Patient 3—HCM and RASopathy, autosomal recessive LZTR1 variants

**Figure 4:**
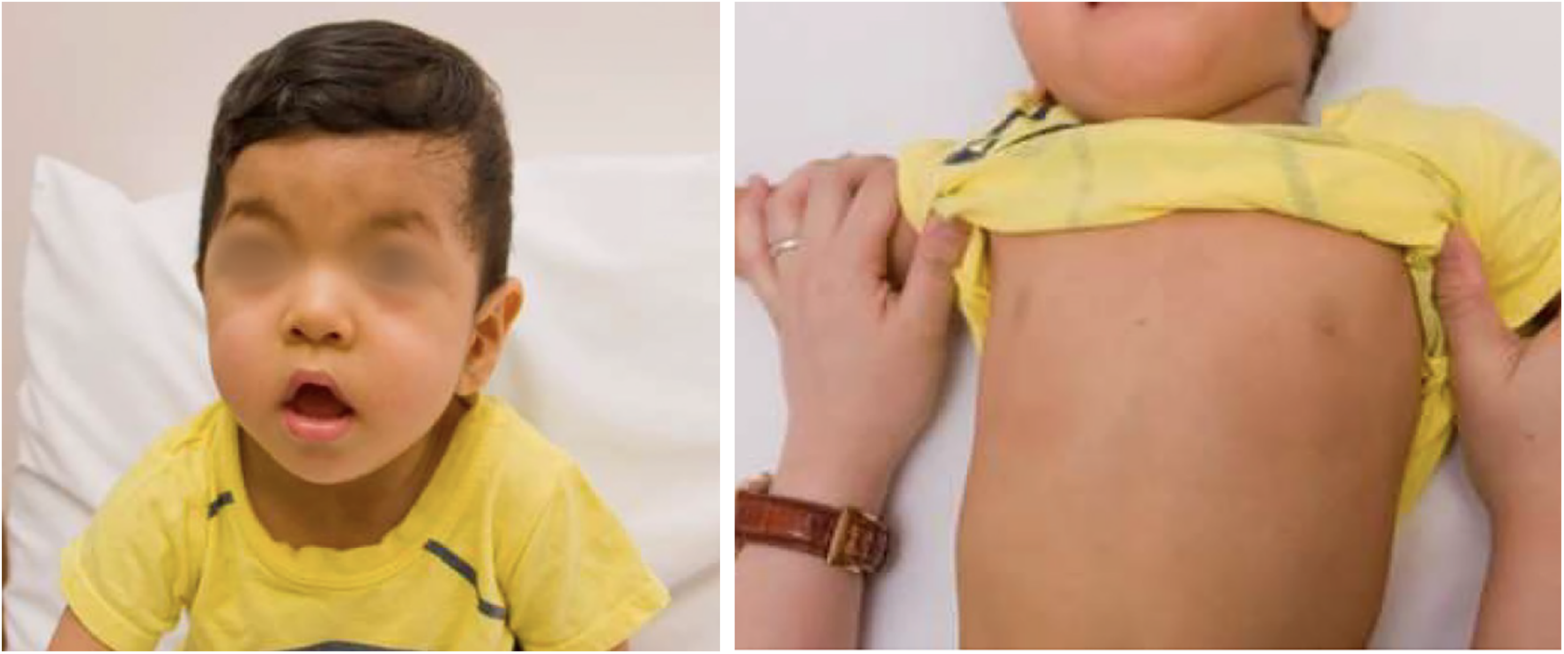
Patient 3 showed clinical features of noonan syndrome: hypertelorism, down-slanting eyes, pectus carinatum.

The patient presented with antenatal ventricular hypertrophy, with subsequent established postnatal diagnosis of HCM, secundum atrial septal defect and left coronary artery fistula to right ventricle. There was symmetrical hypertrophy of his myocardium, which was compatible with HCM changes related to RASopathy. In addition, he also had Abernethy malformation type 1b, with congenital absence of portal vein. Clinical examination revealed facial features consistent with Noonan syndrome (Figure 4), along with developmental delay. Prior genetic testing utilizing trio WES was negative. There was no significant family history.

WGS identified 2 compound heterozygous putative splice-site variants in leucine zipper-like transcriptional regulator 1 (*LZTR1*) gene. *LZTR1* encodes the BTB-kelch superfamily proteins which control various fundamental cellular processes, with a strong association with RASopathy. *LZTR1(NM_006767.4):c.1943-256C>T* was paternally inherited which was rare in GnomAD dataset (allele frequency = 0.00006159). SpliceAI predicted an effect in splicing, specifically a donor gain of 0.24. Functional analysis confirmed the presence of additional RT-PCR products of 583 bp, including a 117-bp alternate exon retained from within intron 16. The resulting cryptic exon contained a premature stop codon, predicting a truncated protein [p.(Tyr741Hisfs*89)]. Previous literature and ClinVar reported several cases of Noonan syndrome associated with this variant, identified in multiple unrelated affected individuals (28–30). Therefore, the variant is classified as pathogenic (PVS1, PM2_supporting, PM3). Another variant in *LZTR1, (NM_006767.4):c1261-3C>G*, was maternally inherited. SpliceAI predicted an effect in splicing (acceptor loss 0.36; acceptor gain 0.62). The variant was rare in GnomAD control population (allele frequency = 0.000008029). RNA sequencing demonstrated that the variant led to aberrant splicing. The variant was detected in trans with a pathogenic variant. The variant was therefore classified as LP (PVS1, PM2_supporting, PM3). Clinical assessment of his parents and younger sister was negative.

#### 3.1.4 Patient 4–6—likely pathogenic variants in exome region for LVNC and CPVT

Two LP variants and one VUS were identified in one LVNC patient and 2 CPVT patients. The LVNC patient was suspected to have mitochondrial disease with a homoplasmic VUS variant [*NC_012920.1 (MT-TK):m.8338A>G*] identified. However, subsequent metabolic investigations yielded unremarkable results, and the clinical course did not align with a mitochondrial disorder. Through WGS, a LP *MYH7 (NM_000257.4):c.1118C>A (p.Ala373Glu)* variant was identified, which was associated with LVNC.

**Figure 5:**
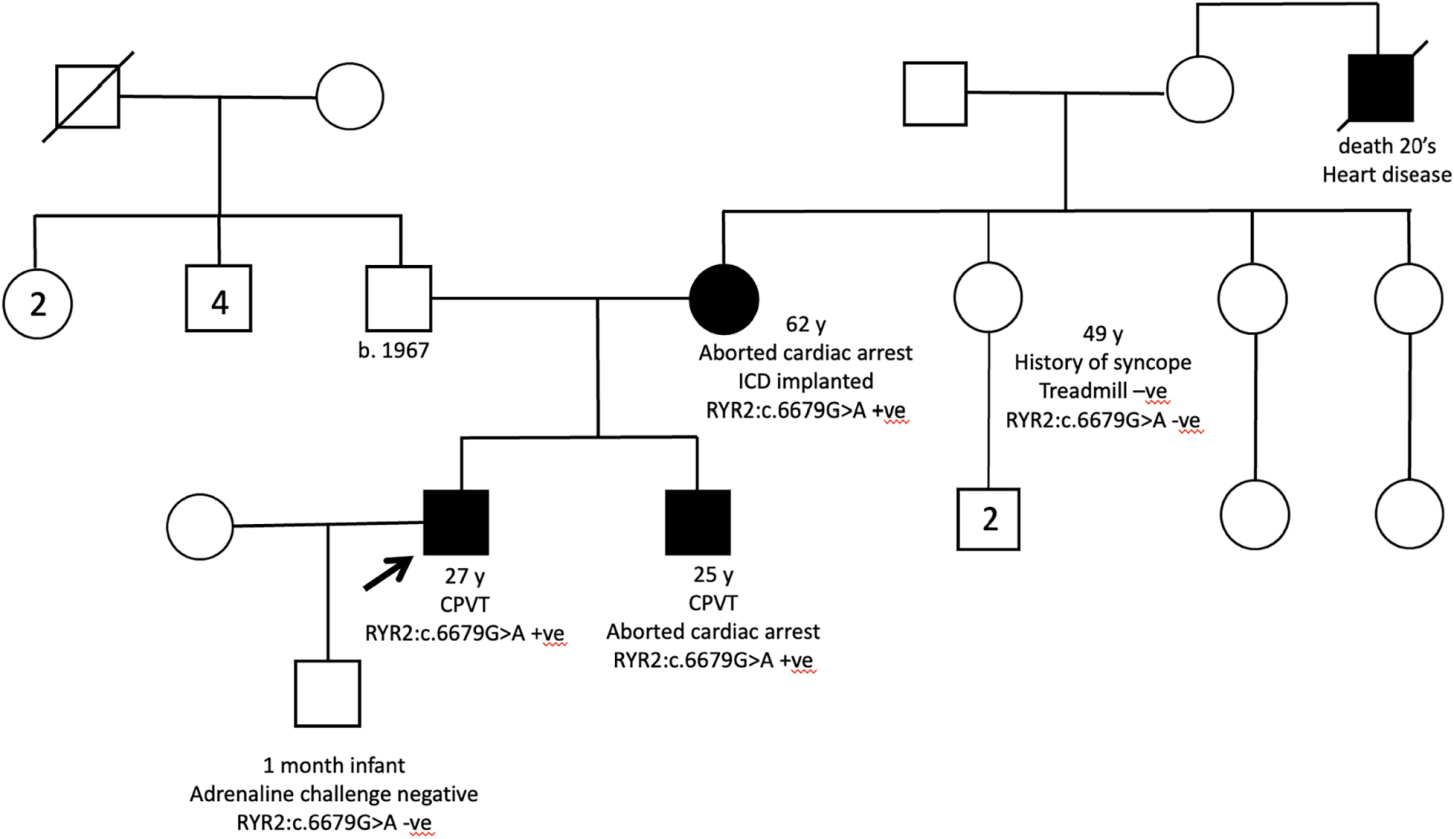
Pedigree of the CPVT family of patient 5.

The probands of two families with CPVT were also recruited with previous negative *RYR2* panel testing. Both probands presented with exertional syncope and positive treadmill test, and one of them had a strong family history of aborted cardiac arrest (Figure 5). Quadruple WGS was performed for the family and a heterozygous *RYR2* variant (*NM_001035.3:c.6679G>A; p.Val2227lle*) was identified in all clinically affected family members. The variant was located in mutation hotspots therefore PM1_strong rule can be applied (31). Singleton WGS was performed for another proband and a heterozygous *RYR2* variant (*NM_001035.3:c.1858T>G; p.Cys620Gly*) was identified as VUS.

#### 3.1.5 Cohort of subjects with prior WES testing

Among the 22 patients who were tested using WES (6 channelopathy and 16 cardiomyopathy), 4 new clinical relevant variants could be identified in 3 cardiomyopathy patients. The use of WGS in this subset of cohort resulted in an additional yield of 13.6%.

## 4 Discussion

Cardiac channelopathy and cardiomyopathy are significant causes for SCD and morbidity in children and adolescents. Through cascade genetic screening of family members, early assessment and intervention can be facilitated. Genetic discoveries also lead to new therapies that target specific disease mechanisms, enabling genotype guided management. This study aimed to demonstrate the use of WGS in a 15-year cohort of paediatric cardiac channelopathy and cardiomyopathy, on top of conventional gene testing. With the expanding use of WGS, this pioneer study serves to share the initial experience of employing this advanced genomic testing in these specific groups. It is worth noting that our study is the first related study on Asian population, and several novel variants are reported. Specifically, four new variants located in intronic regions of genes could be identified in 3 patients.

### 4.1 Extra yield of intronic and splice-site variants using WGS

WGS enables comprehensive scrutiny of single nucleotide variants and small insertions/deletions, within both coding and regulatory regions of the genome. It offers additional coverage at the promoter, intronic regions and the mitochondria genome. Moreover, WGS enables the detection of structural variants which are not possible to identify using panel sequencing or WES.

In recent years, there have been an increase in the identification of disease-causative variants located in deep intronic and mitochondrial regions, some of which can create novel pseudo-exons (32, 33). Identifying and understanding intronic and splice-site variants in paediatric channelopathy and cardiomyopathy are significant because they expand our knowledge of the genetic basis of these conditions. As rare novel variants continue to be identified, RNA sequencing has been developed as a tool to confirm the functional impact of these variants, providing supporting evidence for their pathogenicity. In our cohort, RNA sequencing was used successfully to confirm the functional impact of the *TAFAZZIN* variant in patient 1, and the 2 *LZTR1* variants in patient 3.

WGS allows for a more comprehensive evaluation of the genetic landscape of these disorders, enabling better diagnosis, risk assessment, and potential targeted therapeutic interventions. As novel genes continue to be discovered through WGS, it has transitioned from being a scientific advancement to a clinical diagnostic tool in identifying molecular explanation of cardiac channelopathy and cardiomyopathy. The presence of such variants in affected individuals can also inform genetic counselling, provide insights into disease mechanisms and prognosis, and offer potential treatment options. In our study, the pathogenic intronic variants in the 2 patients identified through WGS enable our patient's families to utilize the variant information for future preconception counselling.

### 4.2 Cost-effectiveness of WGS in paediatric channelopathy and cardiomyopathy

Albeit the discovery of these novel variants, the extra yield using WGS to detect these intronic and splice-site variants was only 1.94%. The low yield was probably due to current limitation of knowledge in these non-exonic variants for cardiac channelopathy and cardiomyopathy, hindering their reporting in the laboratory.

Compared to WES and gene-panel testing, WGS is associated with higher costs, longer turn-around times, and a greater chance of identifying VUS. Additional functional studies, such as RNA sequencing, may also be required to assist determination of pathogenicity of detected variants in these regions, thus adding to overall testing cost. Inevitably, it hinders the use of this advanced genetic methodology currently, especially in resource-scarce settings.

Targeted gene panel testing, on the other hand, balances reasonably comprehensive coverage and are often highly optimized for complete and uniform capture of region of interest. It remains as the first line diagnostic testing for proband in most of cardiovascular units (34). In fact, WES covers all the coding regions and theoretically splicing variant changes in canonical regions near the ends of exon-coding regions can be discovered as well.

Despite variants in these canonical regions should be able to be identified by WES, the limitation of data information in these regions may hinder reporting of these variants. In our study, the variant changes *TAFAZZIN:c.284+5G>A* and *LZTR1:c1261-3C>G* are also located in these areas of interest, which were not reported in pervious WES analysis. Investigations were launched and we could identify the variant change in the initial WES raw data. However, owing to a narrower candidate gene prioritization and more stringent quality control filtering, these variants were not picked up for further analysis in previous WES reporting. As *TAFAZZIN* gene was not included in the core genes for primary cardiomyopathy, the gene prioritization was put at a lower position and the variant change had probably been filtered out. For *LZTR1* gene, the variant change was not reported as the pathogenicity could not be determined with its nature of autosomal recessive inheritance, and without another pathogenic variant detected in-trans in prior WES testing, these *LZTR1* variants might not be reported as well. Therefore, WGS is shown to be superior in the detection of these variants in this region.

This also sheds a light on the alternative approach of re-analysis of sequencing data to look for novel discoveries, before fishing the genotype using WGS. The phenotype determination can also help in the sequencing analysis, especially in pediatric cases in which specific metabolic diseases or autosomal recessive conditions may worth a second inspection or further investigations (27).

Despite the skewed cost-effectiveness in employing WGS in these patients, the importance of genotype-guided management and subsequent cascade family screening may worth the cost. Clinical pictures and family history can definitely help in prioritizing our patient to undergo this advanced genetic testing. For example, patient 1, who exhibited a severe antenatal-onset DCM phenotype with multiple affected male family members, was a good candidate for WGS testing. This made the pre-test probability higher in finding a genetic explanation for the proband. The identification of Barth syndrome in this patient demonstrated the value of WGS. The patient's family benefited from cascade family screening of other maternal siblings, leading to subsequent pre-conception counselling for female carriers. Additionally, WGS could have also guided the possible use of therapies such as elamipretide and gene therapy for refractory heart failure (35–37), although our patient died before receiving a genetic diagnosis.

The balance between cost-effectiveness and clinical utility of WGS will likely shift as the WGS cost decreases and turn-around times improve of in the future. Particularly, with the increasing understanding of intronic, splicing and mitochondrial variants in cardiac channelopathy and cardiomyopathy, it helps clinicians to escape from the potential VUS purgatory. Time is required to gather specific gene-disease and functional data for variants in these mysterious non-coding regions for pathogenicity determination. Until we gain more experience in handling WGS data for cardiac patients, careful patient selection is crucial before subjecting individuals with cardiomyopathy and channelopathy to WGS testing.

Considering the widespread use of WGS, it is extremely important for the interpretation of a cardiac genetic test to be provided by a team of providers with expertise in genetics and cardiology [Class IIA recommendation] (38). A multidisciplinary approach, comprising cardiologists, clinical geneticist, genetic counsellors, genome analysts and bioinformaticians, in an expert centre should therefore be adopted. A scientific statement from the American Heart Association has stressed the importance of the establishment of specialized clinical cardiovascular genetic programs (38). Expertise should be encouraged to be built up in a dedicated centre, especially the classification and interpretation of these genetic information can change over time as new information becomes available based on familial segregation studies and functional validation of genetic variants, resulting in significant differences in up to 18% of genetic results (39).

Despite applying WGS to investigate the molecular basis of clinically irrefutable phenotypes, it still remained gene elusive in some cases of paediatric channelopathy and cardiomyopathy in our cohort. In cardiac channelopathy, out of 77 cases with completion of genetic work-up using WGS, 9 of the cases (11.7%) still remained gene elusive without P/LP variants or clinical relevant VUS. Similarly, in cardiomyopathy, out of 61 cases with completion of genetic work-up using WGS, 16 of the cases (26.2%) still remained gene elusive without P/LP variants or clinical relevant VUS. This suggests that these conditions may not be solely explained by monogenic causes. Given the significant genetic and phenotype heterogeneity, as well as variable penetrance, a polygenic basis of these conditions is now being investigated by genome-wide association studies (GWAS). These studies aim to investigate the role of common variants (with a frequency of >1% in the general population) as protective or predisposing roles by incorporating polygenic risk scores (40–44). Such common variants are routinely screened out by exome or genome based genetic testing. Therefore, in addition to an approach to look for monogenic explanation by escalating searching for disease-causative variants, we should remain open-minded and consider alternative model to better explain the pathogenesis of cardiac channelopathy and cardiomyopathy at molecular level.

### 4.3 Limitation

The study included patients with varying initial genetic testing approaches, ranging from target genetic panel study (sanger sequencing/MLPA/next generation sequencing) to WES. The genes analyzed were selected based on the contemporary genomic knowledge of cardiac channelopathy and cardiomyopathy at the time of genetic testing. There were expected discrepancies in the gene coverage for the panel tests employed our recruited patients, in their old testing. Due to the high cost in old era of genetic testing, either channelopathy or cardiomyopathy gene panels were employed. Initially, we considered repeating another gene panel testing or WES before recruitment into WGS testing. However, given that the diagnostic power of WGS has been proven to be superior to that of gene panel testing, we decided there was no need to validate its diagnostic power by duplicating the gene panel testing prior to recruitment. As for WGS, we used at least 30× depth for detailed analysis. We adjusted our hypothesis to test WGS's ability to identify intronic and spice-site variants in our special cohort. No matter what methodology of testing was employed in prior testing, intronic variants should not be picked up by conventional gene panel testing.

Although direct comparison of WES and WGS cannot be performed under this methodology, if the identified variants are outside the exome, it is logical to assume the inability for WES to detect these variants. In fact, all our intronic and splice-site variants were discovered in subjects with WES performed previously, this further supports our hypothesis that WGS can uncover those variants which were not detected by WES. Furthermore, we illustrate the role of WGS over WES in uncovering intronic variants in cardiomyopathy, but not cardiac channelopathy cases. Cardiomyopathy used to have lower genetic yield in exome-based testing when compared with channelopathy (2–5). Therefore, it is expected that more novel variants outside the coding regions will be discovered with this advanced technology in cardiomyopathy cases.

With regard to the gene coverage used in our WGS analysis, our gene panel list included all genes with definitive and moderate evidence in ClinGen for cardiac channelopathy and cardiomyopathy. Therefore, all the relevant and robust genes were included in the WGS analysis. For genes with disputed or limited evidence, only genes associated with the phenotypes with sufficient evidence were considered. In paediatrics, genes with recessive inheritance are increasingly recognized and discovered (27). Most of these genes were prioritized already for our analysis, except for those genes established as plausible genes after our study period, like *BAG5, CAP2, FLII, KLHL24, LDB3, LEMD2, LMOD2, MYZAP*. As open genome examination was employed in our WGS analysis, ample time was spent to ensure all relevant and updated genes were examined, followed by rectification of evidence necessary to establish the pathogenicity.

Patients with VUS genes of definitive or moderate evidence were not recruited for this WGS study because these relevant genes are likely attributed to the phenotype, although the evidence was not yet sufficient to upgrade those variants to likely pathogenic ones for clinical actions. Therefore, the yield of further advanced genetic test is expected to be low, and values of using WGS in this subgroup may require further studies to address.

Under current methodology, there were still 4 channelopathy and 5 cardiomyopathy patients recruited for WGS without repeating WES. Therefore, 3 variants in exome region in patient 4–6 were included in our report, which should be able to be picked up by contemporary gene panel testing or WES. Those *MYH7* and *RYR2* variants were not identified in prior gene testing because sanger sequencing/multiplex-ligation dependent probe amplification (MLPA) were employed to test certain hotspots in the gene. Therefore, this illustrates the importance of considering repeating testing in patients with a high probability of a specific inherited cardiac disease and a molecular screening performed in a pre-NGS era or with an incomplete NGS panel (31).

Our tertiary paediatric cardiology centre has been at the forefront of genetic testing for cardiac channelopathy cases in our region since 2007. All cases of channelopathy patients should have undergone initial genetic testing, but a few of patients might die before genetic testing can be offered. Therefore, our genetic database should represent almost all cases of paediatric channelopathy in Hong Kong. In terms of paediatric cardiomyopathy, routine genetic testing has only been implemented in the past 5 years. All our active cases were recruited to complete the genetic work-up using WES 2 years before this WGS recruitment (5). Therefore, our cohort may not include older or deceased cardiomyopathy patients who were not tested during the pre-genetic testing era. Finally, the recruitment process faced challenges as well, as a considerable number of patients either refused participation or could not be reached. Despite these difficulties, we were able to recruit 31 patients for WGS analysis.

## 5 Conclusion

The genetic investigation using WGS in our cohort of paediatric cardiac channelopathy and cardiomyopathy had a small additional yield on top of conventional genetic testing. Several novel intronic spice-error variants were identified. WGS can be considered in selected paediatric cases of cardiac channelopathy and cardiomyopathy, especially in gene-elusive proband with a strong phenotype and positive family history. Currently, the widespread use of this advanced genomic technology in cardiac channelopathy and cardiomyopathy is limited, with a cost-effectiveness consideration.

## Data Availability

Original datasets are available in a publicly accessible repository: The original contributions presented in the study are publicly available. This data can be found here: https://www.ncbi.nlm.nih.gov/sra/PRJNA1086226.
