# Supplementary material for: Whole genome sequencing in paediatric channelopathy and cardiomyopathy
Source: Front Cardiovasc Med. 2024 Mar 20;11:1335527. doi: 10.3389/fcvm.2024.1335527 (PMC10997036; doi:10.3389/fcvm.2024.1335527)
Supplement: Supplementary file 1 [file Datasheet1.docx]

Whole Genome Sequencing in Paediatric Channelopathy and Cardiomyopathy

**Supplementary materials**

Supplementary table 1

Variants identified using gene panel testing / WES in prior genetic testing

| Phenotype | Gene identified | Position changes | AF (GnomAD 2.21) | AF (GnomAD 4.0) | Updated Pathogenecity using GnomAD 4.0 |
| --- | --- | --- | --- | --- | --- |
| DCM | *KCNH2* | *c.274C>T* | 0.00005256 | 0.000007476 | VUS |
|  | *CBL* | *c.1484C>T* | 0.00003182 | 0.00002230 | VUS |
|  | *DSP* | *c.2765C>T* | 0.0002029 | 0.0001072 | VUS |
| LVNC | *ACTN2* | *c.1162T>A* | 0.00006721 | 0.00002479 | VUS |
|  | *MT-TK* | *m.8338A>G* | N.A |  |  |
| ACM | *MYOM1* | *c.2991+8 C>A* | 0.0006904 | 0.00001119 | VUS |
|  | *MYH11* | *c.1879G>A* | 0.0001707 | 0.00005168 | VUS |
| Brugada | *AKAP9* | *c.3827G>A* | 0.008861 | 0.007663 | Likely Benign |
| Idiopathic VT/VF | *VCL* | *c.2521G>C* | 0.001138 | 0.0003407 | Likely Benign |
|  | *KCNA5* | *c.156G>T* | 0.00002008 | 0.000008872 | VUS |
|  | *GAA* | *c.1298A>G* | 0.00001157 | 0.000008730 | VUS |

DCM: Dilated cardiomyopathy; LVNC: Left ventricular non-compaction cardiomyopathy; ACM: Arrhythmogenic cardiomyopathy; VT/VF: Ventricular tachycardia/ ventricular fibrillation; VUS: variant of unknown significance

Supplementary Table 2

| Gene panel specific to cardiomyopathy (n=29) curated by the American College of Medical Genetics and Association of Molecular Pathology (ACMG/AMP) | *ACTC1, ACTN2, BAG3, CSRP3, DES, DSC2, DSG2, DSP, GLA, JUP, LAMP2, LMNA, MYBPC3, MYH7, MYL2, MYL3, PKP2, PLN, PRKAG2, RBM20, RYR2, SCN5A, TMEM43, TNNC1, TNNI3, TNNT2, TPM1, TTN, TTR* |
| --- | --- |
| Expanded cardiovascular disease gene panel (n=217) adopted from the United Kingdom National Health Service (NHS), GeneDx, and Blueprint Genetics, which included primary cardiomyopathy and cardiac channelopathy genes | *ABCC9, ACADVL, ACTA1, ACTC1, ACTN2, AKAP9, ALG10, ALG10B, ALMS1, ALPK3, ANK2, ANKRD1, ATAD3A, BAG3, BRAF, CBL, CACNA1C, CACNA1D, CACNA2D1, CACNB2, CALM1, CALM2, CALM3, CALR3, CASQ2, CAV3, CDH2, CLCA2, CMH21, COA6, COX15, CPT2, CRELD1, CRYAB, CSRP3, CTF1, CTNNA3, DES, DMD, DMPK, DNAJC19, DOLK, DPP6, DSC2, DSG2, DSP, DTNA, ELAC2, EMD, EPG5, EYA4, FBXO32, FHOD3, FHL1, FHL2, FKRP, FKTN, FLNC, FXN, GAA, GATA4, GATA5, GATA6, GATAD1, GJA5, GLA, GPD, GPD1L, GYG1, HCN4, HEY4, HFE, HRAS, IDS, IDUA, ILK, JAG1, JPH2, JUP, KCNA5, KCND3, KCNE1, KCNE2, KCNE3, KCNE5, KCNH2, KCNJ2, KCNJ5, KCNJ8, KCNQ1, KLF10, KLHL24, KRAS, LAMA4, LAMP2, LBD3, LMNA, LRRC10, LZTR1, MAP2K1, MAP2K2, MIB1,MRAS, MTO1, MTND1, MTND5, MTTL1, MTTS1, MYBPC3, MYH6, MYH7, MYL2, MYL3, MYL4, MYLK2, MYLK3, MYOM1, MYO6, MYOT, MYOZ2, MYPN, NEBL, NEXN, NF1, NFKB1, NKX2-5, NODAL, NOTCH1,*  *NPC1, NPC2, NPPA, NRAS, NRAP, OBSCN, PCCA, PCCB, PDLIM3, PKP2, PLEC1, PLEKMHM2, PLKHM2, PLN, PPA2, PPCS, PPP1CB, PPP1R13L, PRDM16, PRKAG2, PSEN1, PSEN2, PTPN11, RAF1, RANGRF, RASA2, RBM20, RHBDF1, RIT1, RMB20, RPL3L, RRAS2, RYR2, SCCD, SCN10A, SCN1B, SCN2B, SCN3B, SCN4B, SCN5A, SCO2, SDHA, SDHD, SEMA3A, SHOC2, SGCA, SGCB, SGCD, SLC22A5, SLC25A4, SLC3OA5, SLC40A1, SLC4A3, SLC6A6, SLMAP, SMAD4, SNTA1, SOS1, SOS2, SPERD1, SYNE1, TANGO2, TAZ, TBX20, TBX3, TBX5, TCAP, TECRL, TFR2, TGFB3, TMEM43, TMEM70, TMPO, TNNC1, TNNI3, TNNI3K, TNNT2, TP63, TPM1, TRDN, TRIM63, TRPM4, TTN, TTR, VCL, WRN, ZBTB17, ZIC3, ZNF9* |
| Genes with definitive and moderate evidence under ClinGen: | |
| HCM, DCM | *ACTC1, BAG3, CSRP3, DES, FLNC, FXN, JPH2, LAMP2, LMNA, MYH7, MYL2, MYL3, NEXN, PLN, PRAK2, RBM20, SCN5A, SLC25A4, TNNC1, TNNI3, TNNT2, TPM1, TTN, TTR, VCL* |
| Brugada, CPVT, LQTS, SQTS | *CACNA1C, CALM1, CALM2, CALM3, CASQ2, KCNH2, KCNJ2, KCNQ1, RYR2, SCN5A, SLC4A3, TECRL, TRDN* |
| RASopathy | *BRAF, CBL, HRAS, KRAS, LZTR1, MAP2K1, MAP2K2, MRAS, NRAS, PPP1CB, PTPN11, RAF1, RIT1, RRAS2, SHOC2, SOS1, SOS2, SPRED1* |
